# Resistance to c-Kit inhibitors in melanoma: insights for future therapies

**DOI:** 10.18632/oncoscience.51

**Published:** 2014-06-06

**Authors:** Matteo S. Carlino, Jason R. Todd, Helen Rizos

**Affiliations:** ^1^ Westmead Institute for Cancer Research, The University of Sydney at Westmead Millennium Institute, Westmead, New South Wales, Australia; ^2^ Department of Medical Oncology, Westmead and Blacktown Hospitals, New South Wales, Australia; ^3^ Melanoma Institute Australia, Sydney, New South Wales, Australia; ^4^ Division of Cancer Biology, Institute of Cancer Research, London, United Kingdom; ^5^ Australian School of Advanced Medicine, Macquarie University, New South Wales, Australia

**Keywords:** c-Kit, MAPK, PI3K

## Abstract

Mutations activating the receptor tyrosine kinase c-Kit occur commonly in melanomas arising on mucosal membranes and acral skin. Clinical studies have demonstrated that selective inhibition of c-Kit is effective in treating patients with c-Kit mutant gastrointestinal stromal tumors, but c-Kit inhibitor activity has been disappointing in c-Kit mutant melanoma patients. Activated c-Kit utilises phosphatidylinositol 3-kinase (PI3K) signalling as the dominant effector of cell proliferation and survival with the mitogen-activated protein kinase (MAPK) cascade serving as an ancillary survival pathway. We confirmed that these pathways are re-activated in melanoma cells with acquired resistance to c-Kit inhibitors and that these resistant sublines remain sensitive to the concurrent inhibition of MAPK and PI3K signalling. These findings suggest that durable responses in c-Kit mutant melanoma may require combination therapies that selectively inhibit critical downstream proliferative and survival pathways. We also discuss the interaction between targeted therapies and anti-tumor immune responses and the need to consider immunotherapies in new combinatorial treatment approaches.

The constitutive activation of the c-Kit receptor tyrosine kinase via somatic mutations is uncommon in melanoma (3% are c-Kit mutant), but occurs in approximately 20% of melanomas arising from acral skin (palms, soles and nail bed) or mucosal surfaces and less frequently in cutaneous melanomas found in chronically sun-damaged skin [1]. c-Kit mutations allow for the ligand-independent activation of this receptor and the constitutive downstream activation of the mitogen activated protein kinase (MAPK) and phosphatidylinositol 3-kinase (PI3K) signalling cascades [2] (Figure 1).

**Figure 1:**
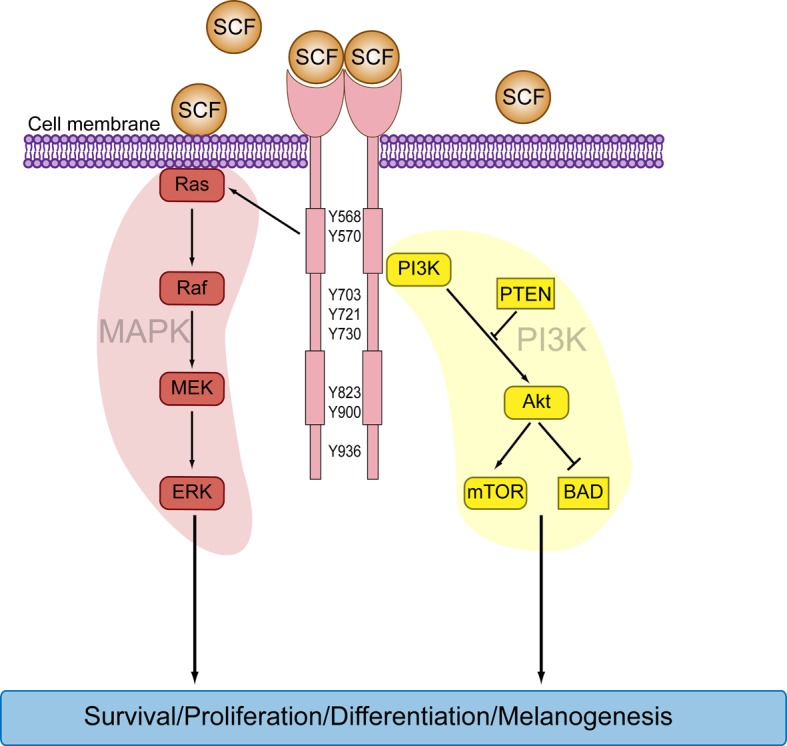
c-Kit signalling activates the MAPK and PI3K signalling cascades Binding of the dimeric ligand SCF triggers the dimerization, phosphorylation and activation of c-Kit. Phosphorylated c-Kit tyrosine residues serve as high-affinity binding sites for signal transduction molecules, which promote the activation of the MAPK and PI3K cascades. The numbers refer to tyrosine residues phosphorylated in c-Kit.

The importance of mutant c-Kit as a therapeutic target has been demonstrated in gastrointestinal stromal tumors (GIST), which frequently harbour activating c-Kit mutations [3]. The treatment of c-Kit mutant GIST with imatinib mesylate, a selective inhibitor targeting c-Kit, Abl and platelet-derived growth factor receptor, produces responses in 80% of patients with over 90% of these patients remaining progression free at one year [4]. In c-Kit-mutant melanoma the response rate to imatinib is only 30% and clinical benefit is transient with median progression free survival of only 3 to 4 months [5-7].

The diminished clinical efficacy of imatinib-based therapy in c-Kit-mutant melanoma patients may reflect the high genetic mutation load found in melanomas and the distribution of activating c-Kit mutations. The average somatic mutation rate of 30 mutations per Mb in three c-Kit mutant melanomas is high compared to other tumors and pre-existing alterations, including activating H-RAS mutations or loss-of-function p16^INK4a^ variants, may diminish c-Kit inhibitor responses [8]. For instance, an acquired activating N-RAS^Q61K^ mutation was associated with c-Kit inhibitor resistance in a c-Kit mutant melanoma [9]. Further, although approximately 70% of c-Kit mutations in melanoma and GIST occur in exon 11, there is a preponderance of the activating L576P, exon 11 variant in melanoma (~34% of c-Kit mutations) and this mutation shows poor imatinib sensitivity in GIST [10], and variable sensitivity in melanoma [5, 7]. Several other c-Kit inhibitors, including sunitinib [9], dasatinib [11] and nilotinib [12] are active in c-Kit mutated melanoma, but their inhibitory profile varies and comparisons with imatinib are difficult due to the small numbers of reported patients on alternative kinase inhibitors.

Targeting signalling effectors downstream of driver oncogenes may be an effective, alternative therapeutic strategy. For instance, the pharmacological inhibition of MEK (the downstream target of BRAF) improves overall survival in patients with BRAF-mutant metastatic melanoma, when compared with chemotherapy [13]. The activation of c-Kit, either via its ligand, stem-cell factor (SCF), or oncogenic mutation activates MAPK and PI3K pathways (Figure 1) and we examined the contribution of these cascades in c-Kit mutant melanoma [14]. We showed that PI3K signalling was the dominant effector of wild-type c-Kit mediated proliferation and migration, and that this pathway remained essential for the proliferation and survival of c-Kit mutant melanomas. Consequently, selective inhibition of PI3K induced proliferative arrest and cell death in c-Kit mutant melanoma cells. Significantly, PI3K inhibition did not replicate imatinib activity in c-Kit mutant melanoma because MAPK signalling was also activated and provided ancillary survival signals in these cell models. Accordingly, the simultaneous inhibition of MAPK and PI3K signalling was required to induce strong synergistic death of c-Kit mutant melanoma cells, with comparable efficacy to that seen with imatinib [14].

The fundamental roles of MAPK and PI3K signalling were also demonstrated in c-Kit mutant melanoma cell models with acquired resistance to c-Kit inhibitors imatinib and nilotinib [15]. Multiple independent mechanisms of resistance developed in the c-KIT^L576P^ mutant M230 melanoma cell line after prolonged exposure to these c-Kit inhibitors. The genetic effectors of resistance included additional secondary c-Kit mutations (A829P or T670I) and c-Kit independent mechanisms [14]. Given multiple resistance mechanisms, with varied responses to alternative c-Kit inhibitors, developed from a single parental cell line it is reasonable to predict that clinical resistance may also be heterogeneous. In GIST, intra- and inter-lesional heterogeneity of resistance mechanisms is common, and multiple, independent secondary c-Kit mutations occur in GIST patients progressing on imatinib therapy [16, 17]. Secondary point mutations associated with imatinib resistance are frequently located in the drug and ATP binding pocket of c-Kit (encoded by exons 13 and 14) or in the activation loop (encoded by exons 17 and 18). Many of these imatinib-resistant second-site c-Kit mutations are also refractory to sunitinib [18], and consequently sequencing of c-Kit inhibitors is unlikely to be clinically useful in treating c-Kit mutant melanomas. In our preclinical studies, the carriage of the secondary c-Kit^A829p^ mutant rendered melanoma cells resistant to imatinib and sunitinib, but these cells remained sensitive to nilotinib and dasatinib. In contrast, sublines with the second-site c-Kit^T670I^ mutation showed resistance to imatinib, nilotinib and dasatinib, but responded to sunitinib. As expected sublines with c-Kit independent mechanisms were resistant to all tested c-Kit inhibitors. Importantly, regardless of the resistance mechanism, all c-Kit inhibitor resistant M230 sublines re-activated MAPK and PI3K signalling and remained sensitive to the concurrent inhibition of these two pathways.

Our work highlights the central role of the PI3K and MAPK cascades in c-Kit mutant melanoma, and the clinical potential of concurrently inhibiting these pathways. This treatment strategy is also being explored in patients with BRAF-mutant melanoma and may prove effective in circumventing acquired drug resistance. Recent reports have confirmed that BRAF-inhibitor treated melanoma patients rapidly develop multiple, independent mechanisms of resistance that can exist within a single progressing tumor [19, 20]. We also confirmed that at least three independent mechanisms of imatinib resistance arose from a single c-Kit mutant melanoma cell line, and although these resistance effectors displayed differing sensitivity profiles to alternative c-Kit inhibitors, all responded to the concurrent inhibition of MAPK and PI3K signalling. These data indicate that selecting an effective second-line therapy requires a comprehensive analysis of resistance mechanisms and their role in activating oncogenic survival pathways.

Recent reports have also demonstrated that the therapeutic efficacy of targeted therapies, including c-Kit and BRAF inhibitors may depend on anti-tumor T-cell responses. For instance, BRAF-mutant melanoma patients treated with BRAF inhibitors exhibit increased tumor infiltration by CD8+ lymphocytes early during therapy [21]. Similarly, the therapeutic effect of imatinib in GIST requires the immune system and responses to the c-Kit inhibitor dasatinib in c-Kit mutant P815 mastocytomas were dependent on CD8+ T cell-mediated immunity [22, 23]. The unexpected immune effects of targeted therapies appear to operate via several mechanisms including increasing tumor cell antigen expression, selectively decreasing regulatory T cells, reducing the immune-suppressive indoleamine 2,3-dioxygenase (IDO) pathway and increasing the function of natural killer cells [22-25]. Combinations of targeted therapy and immunotherapy are ongoing in BRAF-mutant melanoma and currently involve the combination of BRAF inhibitors and ipilimumab, a monoclonal antibody targeting the cytotoxic T lymphocyte antigen-4 inhibitory receptor. The use of imatinib with ipilimumab induced tumor clearance in a spontaneous model of GIST and immune-stimulation strongly synergised with the c-Kit inhibitor dasatinib to eradicate P815 c-Kit mutant mastocytomas in treated mice [22, 23].

Molecular targeted therapies for the treatment of c-Kit mutant melanoma have been disappointing. Clinical responses are likely diminished by pre-existing genetic alterations and early resistance is heterogeneous and frequently involves the re-activation of downstream c-Kit signalling pathways. The current management of c-Kit mutation-positive melanoma patients involves enrolment in clinical trials using a c-Kit inhibitor or immunotherapy, and there is minimal data for efficacy of immune-modulators in c-Kit mutant melanomas [26]. To achieve durable responses, combination therapies simultaneously targeting multiple, independent pathways (i.e. MAPK and PI3K and/or immune-response) should be explored in the setting of treatment naïve c-Kit mutant melanoma patients and patients with acquired resistance to c-Kit inhibitors. The recent successes and durable responses observed with antibodies that block the inhibitory T-cell receptor PD-1 or its ligand in patients with advanced melanoma provides new opportunities for these combinatorial approaches [27].
